# Pre-Cancerous Lesions in the Oral and Maxillofacial Region: A Literature Review with Special Focus on Etiopathogenesis

**Published:** 2016

**Authors:** Soussan Irani

**Affiliations:** 1 *Dental Research Center, Dept. of Oral Pathology, Dental Faculty, Hamadan University, Hamadan, Iran*; 2 *School of Medicine, Griffith University, Queensland, Australia*

**Keywords:** Face, Head, Leukoplakia, Mouth, Neoplasm, Precancerous condition, Skin

## Abstract

Many types of cancers develop in the oral and maxillofacial region. Squamous cell carcinoma is the most common cancer and constitutes over 90 percent of these tumors. Malignant transformation is a genetic process, which later makes a phenotyping change at the cellular level. Some cancers such as oral squamous cell carcinomas (OSCCs) develop from pre-malignant lesions and conditions. Despite advances in the treatment of OSCC, the 5-year survival rate remains approximately 50% due to inability of early detection of OSCC and precursor lesions. Early detection of oral cancer, especially in the premalignant stage, can decrease mortality and morbidity significantly. This article reviews some clinical, histopathological features and etiopathogenesis of pre-cancerous lesions of the oral cavity and skin of face and lip vermilion. A relevant English literature search in Pubmed, Science Direct, and Google Scholar was performed from 1930 to 2015. Full text of 191 articles met the specific inclusion criteria for this review.

## Introduction

Different types of cancer develop in the oral and maxillofacial region. Squamous cell carcinoma is the most common cancer and constitutes over 90% of these tumors. Malignant transformation is a genetic process, which makes a phenotyping change at the cellular level (1). Some cancers such as oral squamous cell carcinomas (OSCCs) develop from pre-malignant lesions and conditions (2). Development of the oral cancer is a multistep process including genetic, epigenetic, and metabolic alterations (3). Despite advances in the treatment of OSCC, the 5-year survival rate remains approximately 50% due to inability of early detection of OSCC and precursor lesions (2). Early detection of a malignancy, especially in the pre-malignant stage, can significantly decrease mortality and morbidity (4). 

From the etiological and clinicopathological aspects, tumors in the oral and maxillofacial region can be divided into two main categories: cancers of the oral cavity and cancers of the skin of face including lip vermilion. 

This article reviews pre-cancerous lesions of the oral cavity and skin of face and lip vermilion with particular emphasis on etiopathogenesis. 

## Methods

A relevant English literature search in PubMed, Science-Direct, and Google Scholar was performed. The keywords; ‘face’, ‘head’ , ‘leukoplakia’, ‘ mouth’, ‘neoplasm’,‘ precancerous condition’, and ‘ skin’ were searched in title/abstract of publications; limited to 1930 to 2015. The inclusion criterion was all related precancerous lesions. A total of 3056 articles were found. Among them, full text of 191 articles met the specific inclusion criteria for this review.

**Oral Cancer**

Oral cancer is considered as the sixth most common cancer worldwide (5). In 96% of cases, the oral cancer is detected above the age of 40 (6). The ratio of males to females is 2:1 but in older ages, the ratio is nearly 1:1.The tongue, particularly the posterior-lateral surface, is the most common site in both men and women (7). Malignant lesions of the floor of mouth, soft palate and oropharynx are also common. These locations are surfaced by an unkeratinized mucosa. It is assumed that carcinogens dissolved in the saliva, have a prolonged contact with a thin mucosal barrier, so have better access to the surface epithelial squamous cells (8).

In terms of etiology, oral cancers have a strong association with tobacco use (9, 10). In addition, alcohol may involve in the tumor development by increasing oral mucosal permeability, which facilitates the passage of carcinogens such as nitrosamines. Additionally, alcohol also has effects on the cell membrane, and inhibits DNA repair (11). Plummer–Vinson syndrome (PVS) also called Paterson–Brown–Kelly syndrome, a type of iron deficiency anemia, contributes to an increased risk of oral cancer as well (12). Recently, human papillomavirus (HPV) has been suggested as another the etiologic factor for the oral and oropharyngeal cancer (13). Early genetic changes at specific chromosome sites (3p14 and 9p21) involve in malignant transformation (14). Studying the gene mutations can help in distinguishing the lesions with a higher risk of progressing to malignancy. For example, allelic loss of either 3p or 9p chromosome arms has been detected in 50% of leukoplakias which is associated with a 3-8 fold increased risk of malignant transformation (15). Moreover, the anterior oral cavity is constantly exposed to chemicals, drinks, food, infectious agents, and physical injury (16). 

**Leukoplakia**

This term often causes confusion and controversy. According to WHO definition, leukoplakia is a white patch or plaque that cannot be characterized clinically or pathologically as any other disease (17). Smokeless tobacco is associated with developing a leukoplakia in 8.4% of cases (18). Clinically, leukoplakia presents in different views including thin, thick or homogeneous, granular or nodular and proliferative verrucous leukoplakia (19). The risk of malignant transformation significantly increases among people aged 60-70 years (20). Leukoplakias on the floor of mouth, lateral tongue, and lower lip show more dysplasia or malignant transformation (20-22). The possible risk factors for malignant transformation are female gender, idiopathic leukoplakia (in non-smokers), larger than 200 mm^2^, long duration, non-hemogenous type, presence of Candida species*, *and epithelial dysplasia (4). Location on the tongue and/or floor of mouth that oral leukoplakia with dysplasia has a higher risk of malignant transformation rate compared to oral leukoplakia without dysplasia (23). Speckled leukoplakia (white lesions or white nodular patches interspersed with erythematous regions) and erosive leukoplakia are often associated with epithelial dysplasia or carcinoma (24, 25).

Different molecular markers have been detected regarding dysplastic changes, and malignant transformation of oral leukoplakia. For example, accumulated p53 protein has been shown in 89% of oral leukoplakias, mainly in basal layers (26). Mutated TP53 in premalignant oral lesions was assumed to predict malignant progression (27). TP53 and Mdm2 are highly expressed in the leukoplakia cancer group compared to the normal group (28). Another study has reported the association between a higher expression of SMAD4 and increased rate of malignant transformation (29). Overexpressionin of cyclin D1 and p63 with increasing severity of dysplasia and also a decrease in p27 expression have been found in oral leukoplakia as well (30). Additionally, increased expression levels of metalloproteinases 1, 9, and 11, and vascular endothelial growth factor (VEGF) have been detected in dysplastic leukoplakia progressing to squamous cell carcinoma compared to those that do not (31, 32). Overexpression of the human telomerase reverse transcriptase (hTERT) associated with increased telomerase activity has been detected in oral leukoplakias as an early phenomenon in the process of carcinogenesis (33). A previous study has shown the overexpression of retinoblastoma (Rb) protein in leukoplakic lesion compared to the normal tissue (34). Increased expression level of COX-2 and Ki-67 are related to the degree of dysplasia (35).

**Fig 1:**
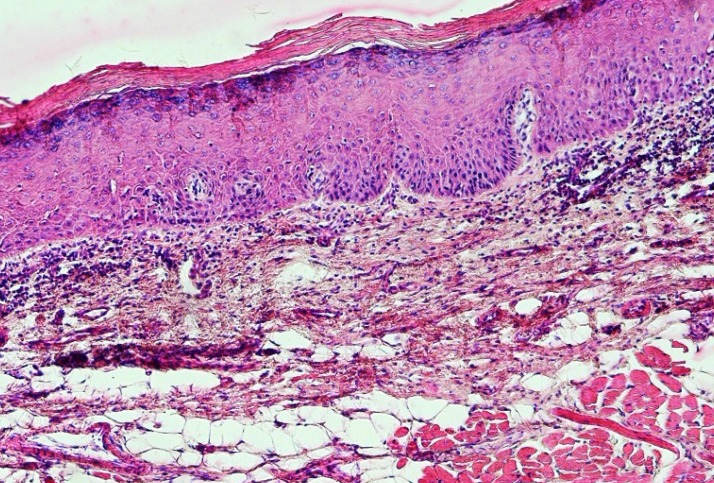
A photomicrograph of a leukoplakic lesion showing mild epithelial dysplasia. (H & E, X 40

Leukoplakia has no specific histopathological feature and is only a clinical term (36). However, histopathologic findings are hyperkeratosis with or without epithelial dysplasia (Figure 1). Epithelial dysplasia is divided into three subclassifications: mild, moderate, and severe (37). Although the presence of epithelial dysplasia is the gold standard for the detection of malignant transformation of the lesions, but there are three major problems as follows: (1) as the diagnosis is subjective it cannot be standardized; (2) not only all lesions with dysplasia do not become a malignant lesion but also some of them even regress; and (3) in some cases carcinoma develops from lesions without any previous history of epithelial dysplasia (38). 

Other oral white lesions such as frictional keratoses, morsicatio buccarum are not considered as leukoplakia as they are not premalignant lesions, and are reversible after elimination of suspected etiological factors. Additionally, other oral white lesions such as candidiasis, lichen planus, leukedema should not be considered as leukoplakia as they have specific microscopic features (19).

**Proliferative verrucous leukoplakia**

Proliferative verrucous leukoplakia (PVL) is a rare lesion. In the early stage it is similar to conventional leukoplakia, both clinically and histopathologically (39), but in the advance stage it appears clinically as verrucous carcinoma (40). PVL is classified as a potentially malignant lesion in the oral cavity (38). In the clinic, the lesion initially develops as a focal hyperkeratosis, which gradually progresses to form an exophytic multifocal lesion (41). Therefore, it is characterized by 4 phases: 1) focal early development; 2) geographic expansion over time; 3) development of a verrucoid/warty appearance; and 4) malignant transformation. In some patients several different OSCCs can develop, therefore, PVL has been considered as a representative of the concept of field cancerization (42). PVL shows variable microscopic features. In early stages, it shows a benign hyperkeratosis. With time, it appears as a papillary and exophytic mass. In later stages the papillary proliferation exhibits downgrowth of well-differentiated squamous epithelium with blunt and broad rete ridges, which invades into the underlying lamina propria. In the final stages the invading epithelium transforms to SCC (43). There are no specific histologic criteria, therefore, diagnosis is based on the histopathologic and cilinical features, along with the behavior (44). TP53 mutaion has not been identified in PVL (18).

**Erythroplakia**

**Fig 2:**
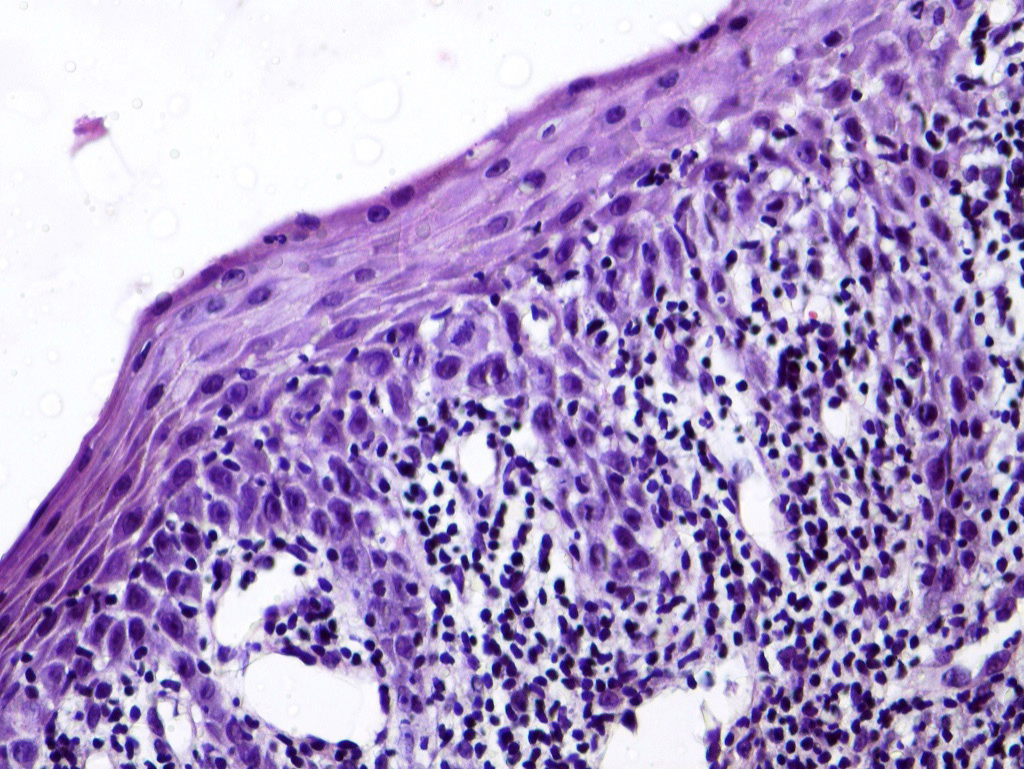
A photomicrograph showing atrophic oral epithelium with atypia, squamatization of the basal cell layer, and underlying chronic inflammation in a clinically diagnosed erythroplakia. (H & E, X 400

Erythroplakia is an uncommon fiery red patch, which cannot be classified as any other condition clinically, and histopathologically (17). Clinically, the lesions present as flat to slightly raised red lesions with irregular borders (40). TP53 mutation has been detected in 46% of oral erythroplakias (45). The histopathological characteristics include the lack of excess surface keratinization, some degree of dysplasia, and even carcinoma in situ or SCC (40, 46) (Figure 2). 

**Verrucous hyperplasia**

 Oral verrucous hyperplasia (OVH) appears as a white or pink single or multifocal plaque or nodule with a verrucous or papillary surface, resembling as a large wart. This term can be used as a clinical or a histopathologic feature (47). Moderate dysplasia is predominant than mild dysplasia and is correlated with consumption of different tobacco preparations (48). Verrucous hyperplasia can develop a malignancy, mostly SCC and in a lesser number a verrucous carcinoma (49). 

**Fig 3:**
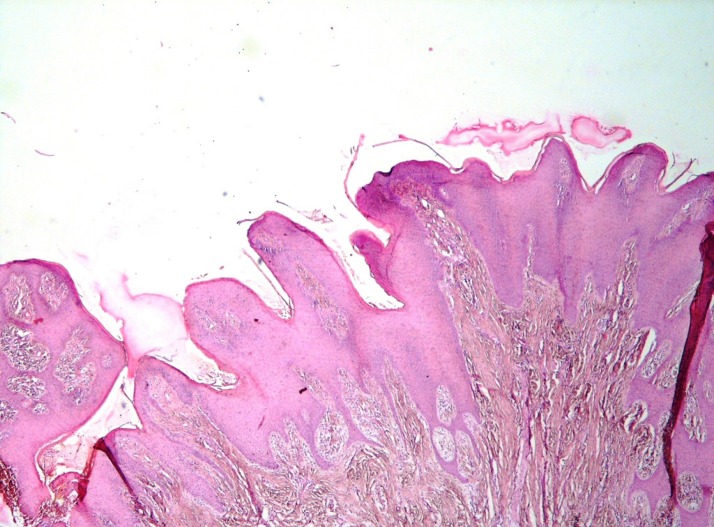
A photomicrograph of a clinically verrucous lesions showing epithelial proliferation. (H & E, X 40

Histopathologic features include sharp and keratotic projections with keratin-filled invaginations without obvious fibrovascular cores (Figure 3). It never extends below that of the adjacent normal epithelium. Mild dysplasia associated with a lichenoid/interface inflammatory reaction can also be seen. In 68% of cases heavy inflammatory cell infiltration including lymphocytes, plasma cells and histiocytes can be observed (50). Lateral and downward growth, broadened and bulbous-like rete ridges are formed. If a broad-front invasion occurs, it can be designated as a verrucous carcinoma. A verrucous carcinoma can be distinguished from a verrucous hyperplasia by a peripheral buttress/shoulder and extension below the lower border of the normal epithelium (51). 

**Tobacco pouch keratosis;** **Smokeless tobacco keratosis; Smokeless tobacco-induced keratos**is;** Snuff dipper’s keratosis**

This lesion is mostly occurs on the buccal or labial vestibule where the tobacco is held, however, the extension of the lesion into the adjacent gingiva and buccal mucosa has been reported (52). In the early stage, it appears as a white wrinkled lesion disappearing by stretching. In the advanced stage, the lesion exhibits as a thickened grayish white zone with folds and fissures. Most of the lesions resolve within 2-6 weeks after cessation of the habit, otherwise, an incisional biopsy should be performed (19). In the microscopic examination, hyperkeratinized and acanthotic epithelium with parakeratin chevrons can be seen. The epithelial dysplasia is not a common finding. Although a significant dysplasia or SCC may be seen, it is usually mild in degree (43). 

**Reverse smoking**

In some countries, due to placing of the lit end of the cigarette or cigar in the mouth, reverse smoking (RS) can develop, and is associated with increased risk of malignant transformation (53). Among 497 patients with leukoplakia, 91.7% of the palatal leukoplakias were found in reverse smokers, and out of 10 oral cancer cases, 9 were located on the palate (54). Keratosis associated with reverse smoking is a precancerous lesion (55). 

Histopathological findings include marked hyperorthokeratosis in 80% of cases associated with epithelial hyperplasia in 73.1% of cases. The granular cell layer is dispersed throughout the upper half of the epithelium. The presence of melanin-containing cells in the basal layer is another histological feature. Mild inflammation can be found in the lamina propria of the palatal biopsies (54, 56). 

**Oral submucous fibrosis**

Oral submucous fibrosis (OSF) is a chronic lesion, which mostly develops in Indians (57). The possible mechanisms in the development of the lesion are increased collagen synthesis or reduced collagen degradation (58). Areca nut contact with epithelial cells induces transforming growth factor beta** (**TGF-β) signaling, which in turn induces inflammation and fibrosis in the underlying connective tissue. In addition, TGF-β produced by epithelial cells can diffuse into the connective tissue (59). The characteristic clinical features of OSF are burning sensation, blanching and stiffening of the oral mucosa such as the lips, tongue, and palate (58). A previous study has indicated that among 371 patients with oral cancer, 30% had OSF. Additionally, the patients with both oral cancer and OSF were younger than patients with oral cancer (45.11 vs 50.07 yr). Oral cancer with OSF was also more common in men (male: female ratio= 10:1) compared with oral cancer (male to female ratio=3.2:1). The tongue was the most common site of involvement in oral cancer-OSF group (60). Up-regulation of some cytokines such as IL-8, IL-6, IL-1, and fibroblast growth factor (FGF) has been reported in fibrosis or OSF (61). In addition, loss of heterozygosity in 23 “hotspot” loci, which controls the cell cycle has been recognized as a malignancy marker in OSF (62). Overexpression of p53 and p63 has been detected in OSF. In a study on PCNA expression status, positive expression of PCNA mainly in basal and suprabasal layers had been detected in all cases of OSF. Although, there was no statistical significant mean difference of PCNA expression in basal and suprabasal layers between OSCC and OSF, there was a statistical significant mean difference in PCNA expression in superficial layers (63). 

In early stages of OSF, the microscopic examination shows a juxtra –epithelial inflammation, followed by hyalinization. Later, the atrophy of epithelium with focal para-keratosis or hyperkeratosis along with imbalance between degradation and synthesis of extracellular matrix (ECM), mainly collagen occurs. Finally, marked collagen accumulation in the lamina propria, submucosa, and superficial muscle layer can be seen (58, 64). Increased deposition of type I collagen, elevated expression of plasminogen activator inhibitot-1 (PAI-1), and tissue inhibitor metalloproteinase-1 (TIMPs) had been show in the connective tissue (65, 66). 

**Oral Lichen Planus and Oral Lichenoid Reaction**

Oral Lichen Planus (OLP) is a chronic inflammatory disease (67). It is suggested that OLP is a T cell –mediated autoimmune disease. Induction of apoptosis of the basal cells of epithelium by CD8^+^ T cells is the possible mechanism of developing of OLP (68). WHO considers OLP as a precancerous lesion especially in the presence of dysplasia (69). Krutchkoff et al*.* criticized this opinion. According to their review, there is not sufficient document in terms of their criteria for the malignant transformation of OLP (70). Krutchkoff and Eisenberg have suggested the term lichenoid dysplasia for cases of OLP with dysplasia. They believed that some reported cases of OLP, which developed a malignancy, were lichenoid lesions with dysplasia (71, 72). These authors proposed histopathological and/or clinico-pathological diagnostic criteria; however, these criteria have not been validated (73). Additionally, both OLP and OSCC are not rare diseases; therefore, they may develop simultaneously (43). On the other hand, there are some reports of developing a malignancy in the same location of previously diagnosed as OLP (74-76). Further, strict clinical studies need to resolve the question.

*Candida albicans, *Hepatitis C virus (HCV) infection, and immunosuppression are considered as the possible risk factors in OLP malignant transformation. Besides, *H. pylori* was detected in 59.2% of OLP tissue samples in a previous study (77). Treatment with topical corticosteroids is also associated with a higher risk of developing a cancer on the OLP lesion (78). Different sites of the oral cavity have been reported as the preferred site for malignant transformation. While some studies have reported the tongue as the preferred site of malignancy (75), some others had indicated the midline of the palate, gingiva and lips (79, 80). The buccal mucosa had been reported as the highest risk site for malignant transformation (81). Interestingly, development of a second carcinoma has been indicated in 50% of the cases among them new malignancy develops in the same site of the primary tumor in 20% of the cases (82). Atrophic-erosive forms were predisposed to cancer development (79, 81), however, in some series, keratotic form (plaque) was more likely to undergo malignant transformation (75, 79, 80). Most cases of OSCC have been reported on the lateral side of tongue, however, some cases of OSCC have been found on the dorsum of tongue (75, 83). 

**Fig 4:**
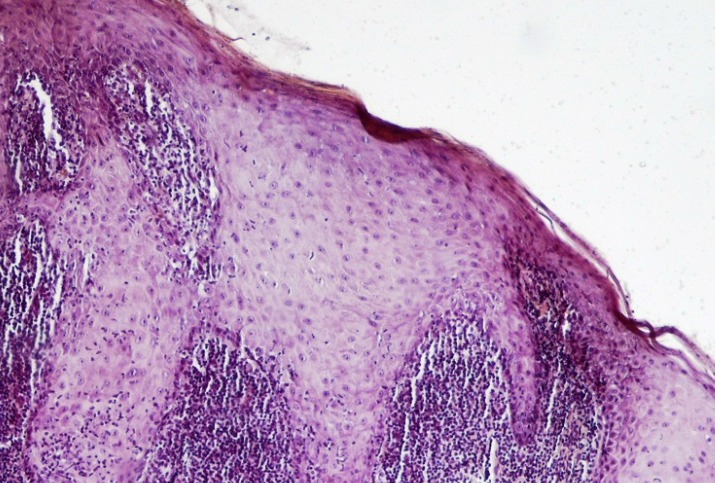
A photomicrograph of a lichen planus lesion showing acanthosis, saw-toothed-shaped rete ridges and band-like infiltration of lymphocytes immediately underlying the epithelium. (H & E, X 400

The presence of a well-defined band-like infiltration of inflammatory cells dominantly lymphocytes, hydropic degeneration of epithelial basal layer, and absence of epithelial dysplasia (Figure 4) are the histopathological criteria for OLP diagnosis (84). The lesions with epithelial dysplasia should not be considered as an OLP lesion. Therefore, terms such as OLP with atypia or OLP with dysplasia should not be used. On the other hand, it is not so easy to rule out the development of epithelial dysplasia in OLP, hence the exclusion of all lesions resembling OLP with epithelial dysplasia may lead the underestimation of malignant transformation rate of OLPs (85). Infiltration of chronic inflammatory cells can be a strong risk factor for cancer development (86, 87). Inflammatory cells may produce an excess nitric oxide (NO). In addition, epithelial apoptosis, probably due to infiltration of inflammatory cells is another risk factor (88), as increased cell proliferation rate of basal epithelial cells results in cancer development (89). A previous study revealed the decreased expression levels of β-catenin, E-cadherin and EGFR in OLP compared to normal tissue (90). Additionally, down-regulation of ANXA1 protein expression was identified in OPLs compared to normal group (91). Oral Lichenoid Reactions (Lesions) (OLRs) are lesions similar to OLPs with different etiology (92). On the lateral border of the tongue, dental materials such as amalgam and composite restorations may be associated with OLR (93). Graft–vs-host disease, seen mainly in bone marrow transplant recipients, is another lichenoid reaction with the potential of developing an oral cancer. A systematic review on the malignant transformation of OLP and OLR found that 85 cases of SCC in developed in OLP lesions and 4 cases of SCC arose in OLRs. Malignant transformation rate for OLP was between 0 and 3.5% and that for OLR was 3.2% (76). A previous study detected the TP53 and Ki67 proteins in OLP and OLR in more than 80% of the cells (94). 

**Lichenoid dysplasia**

**Fig 5:**
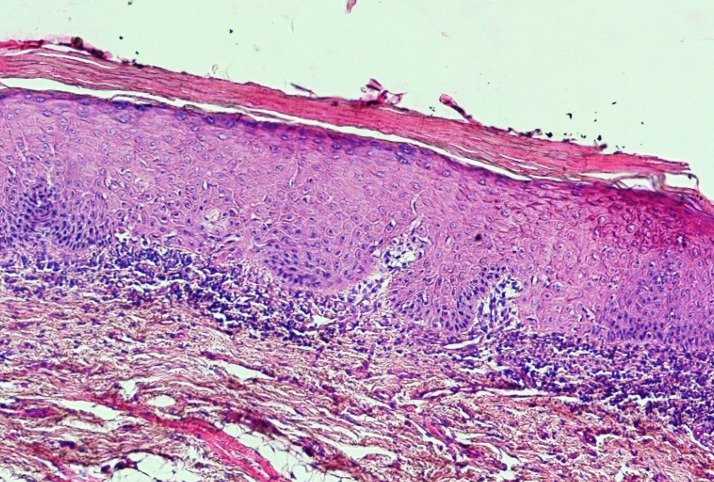
A photomicrograph showing mild epithelial dysplasia, surface hyperorthokeratosis, and lichenoid mucositis. (H & E, X 40

The term lichenoid dysplasia (LD) was introduced in 1985, used in cases of lichenoid stomatitis with dysplasia. Etiopathogenesis of LD is different from that of OLP. In OLP, lichenoid infiltration represents cell-mediated immune response provoked by different antigens, whereas in LD, lichenoid infiltration occurs against atypical epithelial cells (71). Lichenoid dysplasia mostly appears as an erythematous or leukoplakic area on the buccal mucosa or gingiva and is not a symmetrical lesion as can be found in OLP. Microscopic findings of these lesions consist of hyperparakeratosis or hyperorthokeratosis, epithelial dysplasia and band-like lymphocyte infiltration (Figure 5). The basal cell hyperplasia and atypia rather than degeneration is the important histological feature (95). Lack of liquefaction degeneration and intact or even hyperplastic basal cell layer is a major distinguishing characteristic of LD from OLP (71, 96).

**Epidermolysis bullosa**

Epidermolysis bullosa (EB) is a heterogeneous group of inherited diseases, characterized by trauma-induced blistering of the skin and mucous membranes (97). Three major EB types are simplex, junctional and dystrophic (98). Infants with EB have generalized recurrent blistering, resulting in ulceration, pseudosyndactyly with mitten-like deformities of hands and feet, nail loss, as well as scarring or strictures of the oral mucous membrane, and esophagus (99). Oral lesions have been reported in the junctional and dystrophic forms (100). Although malignancy mostly occurs on the skin, it can also occur on the oral cavity, especially the lingual mucosa (43, 101, 102).

**Chronic Discoid Lupus Erythematous**

**Fig 6:**
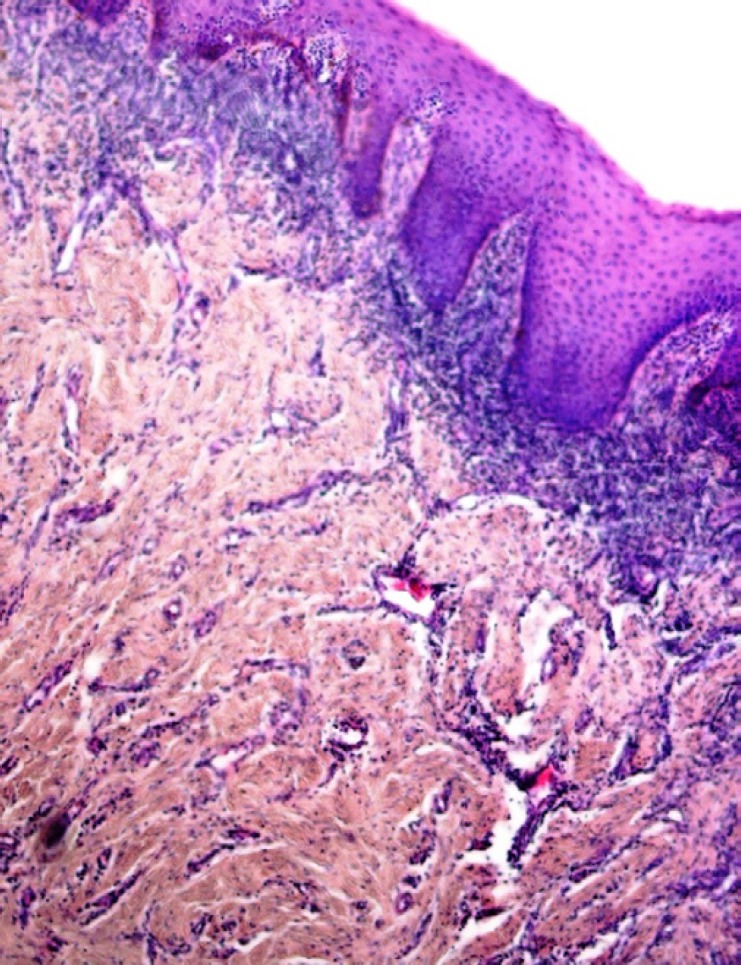
A photomicrograph of a chronic discoid lupus erythematosus lesion showing hyperparakeratosis and lichenoid pattern of inflammation. (H & E, X 40

Chronic Discoid Lupus Erythematous **(C**DLE) is a chronic form of cutaneous lupus, which clinically presents as an erythematous, scaly and depigmented plaque (103). Head and neck area is affected in 41% of all cases (104). Oral lesions are asymmetrically distributed affecting the palate, buccal mucosa and tongue. The buccal mucosa can be affected in 15% of the patients and may transform to leukoplakia (105). The microscopic features include hyperkeratosis, degeneration of the basal layer, and subepithelial lymphocytic infiltration. Deep inflammatory infiltration, often perivascular orientation distinguishes CDLE lesions from OLPs (43) (Figure 6). 

**Dyskeratosis Congenita**

Dyskeratosis Congenita (DC) is a rare inherited bone marrow failure syndrome characterized by the triad of oral leukoplakia (80%), dystrophy of nails (90%), and reticular skin pigmentation (90%) (106). Mutations have been identified in TERC (telomerase RNA component), which provide a direct link between DC, telomerase, and DKC1 gene (107, 108). Malignancies develop in 10% of patients, typically in the third decade of life. The most common malignancy is SCC, and typically develops in areas of leukoplakia (109). Microscopically, oral leukoplakia in DC shows progression from hyperkeratosis to dysplasia (110). Table 1 summarizes the main characteristics of the precancerous lesions of the oral mucosa.

**Table 1:** The main characteristics of the precancerous lesions of the oral mucosa

Lesion	Gender	Age	The most prevalent site	The incidence of dysplasia or /and malignant transformation	The risk factors and possible etiological factors
Leukoplakia	Both	> 50 years	Buccal mucosa, alveolar mucosa, lower lip	5%-19.9%	Smoking, smokeless tobacco, HPV, Candida species
Proliferative verrucous leukoplakia	F	>60 years	Buccal mucosa, tongue	40-100%	Not clear may be HPV and EBV
Erythroplakia	M	>60 years	Floor of the mouth, lateral tongue, retromolar pad	14-67%	Chewing tobacco, alcohol, smokeless tobacco
Verrucous hyperplasia	M	40 years	Buccal mucosa ,tongue	3-17%	Smokeless tobacco, cigarette smoking
Tobacco pouch keratosis	M	Any age	Buccal or labial vestibule	0.6-2.8%	Smokeless tobacco
Oral submucous fibrosis	Both	20-30 years	Fbuccal mucosa	7-30%	Chewing areca and betel quid
Oral lichen planus	F	Middle age	Buccal mucosa, tongue, gingiva	0.4-5.6%	T-cell–mediated autoimmune disease
Oral lichenoid reaction	F	Middle and older	Buccal mucosa, tongue	0.71%	Dental materials
Lichenoid Dysplasis	No data	No data	Buccal mucosa, gingiva	100%	Previous leukoplakia or erythroplakia
Epidermolysis bullosa	Both	Infants	Gingiva, buccal mucosa	Infrequently	Heredity
Chronic Discoid Lupus Erythematous	F	41 years	Palate, buccal mucosa and tongue	13.64%, 0.5-2%	Sun exposure
Dyskeratosis Congenita	Both	10 years	Tongue,buccal mucosa	35%	Mutation of TERC gene

**The Facial and Vermilion Lip Cancer**

**Actinic cheilitis**

Actinic cheilitis (AC) is a chronic inflammatory lesion (111). In the clinical examination, the lesion is characterized by the darkening of the lip and atrophy of the vermilion border at the borders. Over time, scaly areas develop and become thick by extending to the wet line of the lip. Chronic focal ulcers as well as leukoplakic lesions can occur (112). AC may transform into SCC (112), but SCC arising on AC rarely metastasizes to cervical lymph nodes. A malignancy develops in patients older than 50 years of age who use tobacco and are exposed to the sun chronically (113). 

Histopathologically, AC presents a variety of changes including varying degrees of keratosis, epithelial hyperplasia or atrophy, solar elastosis, and the presence or absence of dysplasia (111). The most important aspect is the keratinocyte atypia, which gradually occurs in the epithelium. The number of mast cells increases compared to normal samples (114). Mast cells play a crucial role in inflammation, and contribute to the defense against tumor development as well as its invasion (115,116). However, some previous studies indicated that mast cells could promote ECM degradation and tumor progression (114). CD1a-positive Langerhans cells and mast cells were found in the lamina propria and epithelium of AC, respectively. CD1a-positive Langerhans cells were assumed to have a protective role against transforming into SCC, but the role of mast cells in AC has not yet been defined (117). 

**Actinic keratosis**

Actinic keratosis (AK) is a cutaneous neoplasm composed of transformed keratinocytes as the result of chronic UV exposure (118), specifically; UV-B radiation which causes mutation in the p53 gene. Other etiological factors include fair skin, light colored eyes, male gender, older age, and increased sun exposure (119). Many of the AK lesions are asymptomatic, usually as an erythematous papules or plaques on sun-exposed areas. It may regress or progress to an invasive SCC. AK is the early form of squamous cell carcinoma in situ (120). Some risk factors have been known such as skin type, the amount of photo damage and a history of immunosuppression (121). Some clinical features such as induration, inflammation, diameter larger than 1cm, rapid enlargement, bleeding, erythema and ulceration suggest an increased risk for malignant transformation (122). The length of time to progress to SCC is 24.6 months (123), and the rate of metastasis is quite low, only in 1–2% of cases (124). The strong expression of Keratin-14 in spinous and granular layers of SCC tissue developed from AK, is probably a prognostic factor for tumor progression of AK (125).

**Fig 7:**
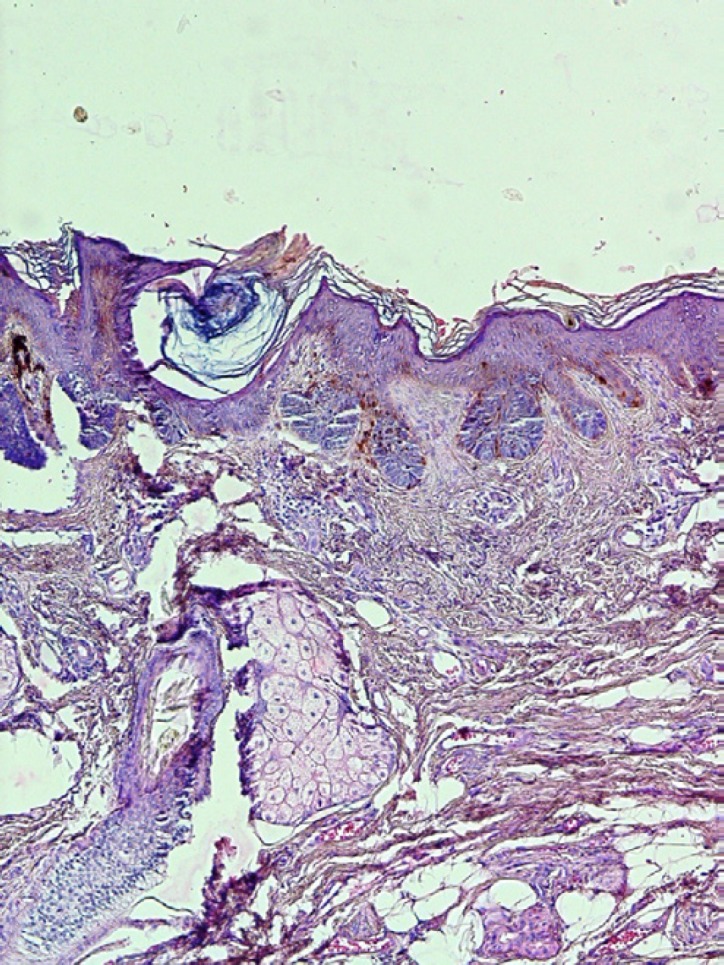
A photomicrograph of an actinic keratosis showing surface hyperkeratosis and acanthosis. (H & E, X 40

The histopathological features of AK include an atrophic or acanthotic or even normal thickness of the epidermis. The acanthotic variant is characterized by elongated rete ridges. Atypical keratinocyte is the pathognomonic feature, which begins within the basal layer cells. In advanced stages, keratinocyte atypia extends above the basal layer (81) (Figure 7). Elastosis formation associated with the infiltration of mast cells can be seen in AK lesions. It has been postulated that mast cells stimulate elastosis via activation of fibroblasts to secrete elastin and proteases such as matrix metalloproteinase (MMP) (126). In human both ultraviolet and infrared radiation may stimulate mast cell proliferation. In addition, keratinocytes or fibroblasts can produce chemotactic factors for mast cells (127). The number of T–cells and Langerhans cells significantly increases in inflamed AK and decreases by progression to SCC. These findings may indicate that progression changes from benign to malignant lesions are associated with an inflammatory response (128).

**Epidermolysis bullosa**

Developing a malignancy within chronic skin wounds and long-term scars is very common event in recessive dystrophic EB. Scarring and repetitive tissue stress lead to malignant changes in EB patients. Development of SCC is correlated to the severity and extent of ulceration and scarring (97). A diminished immunological status due to malnourishment can be considered as the pathogenesis in junctional EB cases (129). The patients with severe dystrophic type are at risk of developing a malignancy at younger age than junctional type. The most common age of developing SCC for dystrophic type is 20-25 years and for junctional type is 28-70 years. For both types, the patients have the risk of developing multiple primary SCCs (97). Mutation of p53 has been detected in EB related SCC (130). Mutation of the *COL7A1 *gene, encoding type VII collagen, has been suggested as the etiologic factor for dystrophic form of the disease (131). Large melanocytic nevi are other findings in EB, which arise in the sites of previous bullae or erosions. These lesions have been reported in children and are susceptible to develop melanoma (132). The risk of melanoma has been reported in patients with the recessive dystrophic form (97). There is also an increased risk of occurrence of multiple keratoacanthomas (133), and squamous cell carcinomas in individuals with junctional EB (134). 

**Chronic Discoid Lupus Erythematous (CDLE)**

SCC and less commonly BCC arise from lesions of CDLE. The mean interval between initiation of the DLE lesion to the appearance of SCC is 30.8 years (135). A long history of DLE in association with bleeding and ulceration of nodules can be considered as progression to SCC (136). Ultraviolet light or radiation used for treatment of DLE in the early 20th century was the etiological factor for malignant transformation (137). Hyperkeratosis, and follicular plugging, vacuolar degeneration of the basal cell layer of epidermis and patchy dermal lymphocytic infiltration are the histopathological characteristics of CDLE (138).

**Chronic Inflammation**

There are some reports of SCC developing in areas of cicatrizing dermatoses, such as Marjolin’s ulcer, and inherited dermatoses like epidermolysis bullosa (139,140). Chronic irritation and infection, and repeated trauma are suggested as etiological factors. Therefore, repeated healing, and toxins released by the damaged tissue may cause the cell mutation (141,142).

Some other precancerous skin lesions which develop a malignant melanoma are as follows:

**Dysplastic Nevi**

Dysplastic Nevi (DN) is clinically acquired melanocytic lesions similar to malignant melanoma. Patients with DN have multiple moles but the majorities are not dysplastic (143). Some of the genetic mutations such as CDKN2A and CDK4 may play role in the pathogenesis of DN (144, 145). DN lesions are associated with overexpression of pheomelanin, which may cause DNA damage and tumour progression (146). Junctional proliferation of single nests of melanocytes often with bridging between nests, melanocytes with large pleomorphic nuclei and lymphohistiocytic infiltrate within the epidermis are histopathological characteristics of DN (147). 

**Congenital Melanocytic Nevus**

Congenital Melanocytic Nevus (CMN) is one of the most common lesions in newborn infant (148). In the clinic, they are round to oval shaped lesions with brown to dark brown color (149). The cause of CMN is not clear but defects in the migration or maturation of melanocytes in the embryo are hypothesized. Characteristic histopathology includes the infiltration of melanocytes into the reticular dermis, and around skin appendages such as follicles and sweat glands. The surgical removal of CMN decreases the risk for development of melanoma (148).

**Nevous sebaceous (of Jadassohn)**

 Nevous sebaceous (of Jadassohn) is a hamartoma of the epidermis and presents from birth which has the potential to develop to BCC. Nearly all Nevous sebaceous (of Jadassohn) can be found on scalp, forehead or face. Clinically, the lesions appear as smooth yellowish, hairless patches (150). Prevention excision is suggested (151). Table 2 summarizes the main characteristics of the precancerous lesions of the skin of the face and lip.

**Table 2:** The main characteristics of the precancerous lesions of the skin of the face and lip

Lesion	Gender	Age	The most prevalent site	The incidence of dysplasia or /and malignant transformation	The risk factors and possible etiological factors
Actinic cheilitis	M	Middle age	Lower lip	62.07%,16.9%	UV, tobacco, alcohol
Actinic keratosis	Both	>40 years	Hand, wrist, and arm	Up to 20%	UV
Epidermolysis bullosa	Both	Infants	Exteremities	76.5%	Heredity
Chronic Discoid Lupus Erythemato	F	41 years	Scalp, ears, lips and nose	3.3%	Sun exposure
Dysplastic Nevi	Both	30-40 years	Scalp,breast and buttocks	5.7-19.7%	Genetic mutation, environmental factors
Congenital Melanocytic Nevi	Both	Infants	Mouth, palms and soles	04-10%	Congenial

## Discussion

Recently, tumor progression models have been made for a few tumors. Genetic pathway correlation helps to construct these models. Not always a benign squamous hyperplasia progresses to a malignancy, therefore, some genetic alterations develop cancers (152). Identification of early genetic alterations, tumor suppressor genes and proto-oncogenes provides necessary information in cancer treatment. Close observation of cases with dysplasia/neoplasia has an impact on patient’s life but there is always a limitation due to the clinical differences between inflammatory benign lesions and true dysplastic/neoplastic changes. Slaughter proposed the concept of field cancerization in 1953 (153). According to this hypothesis, the entire epithelium of upper aerodigestive tract is exposed to carcinogens, therefore, there is a higher incidence of multiple genetic alterations to cause cancer development. In the oral cavity, some etiological factors have been identified. Tobacco smoking and alcohol consumption play important roles in oral cancer. Components of cigarette smoke, including nicotine can stimulate the proliferation of various normal and cancerous cells (154) by increasing the levels of both growth factors such as VEGF, VEGF-C, TGF-b, and growth factor receptors like VEGFR-2, PDGFR, HGFR and EGFR. Nicotine also has anti-apoptotic effect via activation of PKC, PKA and NFkB, and down-regulation of the p53 tumor suppressor protein (155). In a study, elevated dysfunctional p53 was found in heavy smokers. Moreover, *Ras (Rat sarcoma) *mutation has been demonstrated in tobacco chewers (156). Ethanol becomes oxidized into acetaldehyde, which is a carcinogen. Marked levels of acetaldehyde can be detected in saliva after taking ethanol. The oral microbiota may contribute in cancer development due to acetaldehyde production (157). For example, *Candida albicans* has been found in the histological sections of leukoplakia invading the upper epithelium. Variants of human papillomaviruses 16 and 18 are important co-factors, especially in cancers of the tonsils (158). HPV infection inhibits p53 tumor suppressor gene expression, the most well studied mutated gene in the oral pre-malignant lesions (159). Angiogenesis has a pivotal role in carcinogenesis. VEGF is not the only factor involved in angiogenesis. Other factors such as ET axis, Galactin-1 and -3 also contribute in the control of angiogenesis and growth of cancer (86,87, 160). Angiogenesis has been detected in oral pre-malignant lesions, which persist during progression of carcinogenesis. Increased level of VEGF was found in oral pre-malignant conditions (161). Hyperactivity of the EGFR/ERK, and PI3K/AKT/mTOR signaling pathways has been found in OSCC and premalignant cell lines (162). Amplification of proto-oncogene, *cyclin D1*, is another finding in head and neck squamous cell carcinoma (HNSCC) (156). Laminin-5ᵧ2 positivity distinguishes truly oral premalignant lesions from those that are not (163). Podoplanin expression can be used as a predictor of the risk of cancer development in oral precancerous lesions (164).

Skin cancer is the most common human cancer. Basal cell carcinoma (BCC) is the most common skin cancer and the most frequently diagnosed cancer in humans. While BCCs are believed to arise de novo, cutaneous SCCs arise via a multistep process. Keratinocytes gradually acquire new phenotypic characteristics, which lead to aggressive behavior. Molecular and cytogenetic studies on AK provide supporting evidence that AK is a precursor of SCC. Preventing skin cancer with early diagnosis of precancerous skin lesions has a great impact on cancer treatment. Chronic exposure to UV radiation is the major etiologic factor. UV radiation causes p53 genetic mutation, which is a key regulatory molecule in the cellular response to UV radiation. UV radiation leads to migration of Langerhans cells to the draining lymph node, thereby reducing Langerhans cell number in the skin. UV radiation also inhibits the response of mast cells, cytotoxic T cells, and memory T cells. DNA of HPVs has been found in 52% of BCCs, and 41% of AKs (159) which may be another etiologic factor.

## Conclusion

Oral cancer and skin cancer are very common with high mortality rate, therefore, the knowledge about precancerous lesions and their behavior has a crucial impact on patients’ life. Extra oral and intraoral examination of the head and neck region of the patients has a crucial impact on identifying changes affecting the oral mucosa and skin. Careful observation of the clinical and histological changes, along with diagnosis at earlier stages can give lower morbidity and mortality.
